# Prostate cancer screening knowledge and attitude among men over 50 at a referral Hospital in Oshana region, Namibia

**DOI:** 10.4102/jphia.v16i1.652

**Published:** 2025-01-22

**Authors:** Lonia Kashihakumwa, Daniel O. Ashipala, Yahaya Jafaru

**Affiliations:** 1 Department of General Nursing Sciences, School of Nursing and Public Health, Faculty of Health Sciences and Veterinary Medicine, University of Namibia, Rundu, Namibia; 2 Department of Nursing Science, College of Health Sciences, Federal University Birnin-Kebbi, Kebbi State, Nigeria

**Keywords:** attitude, cancer, knowledge, men, prostate

## Abstract

**Background:**

Prostate cancer is the third most common cancer in men and fourth in causing cancer-related deaths in both men and women in Africa.

**Aim:**

The aim of this study was to assess knowledge and attitudes about prostate cancer screening among men over 50 years.

**Setting:**

The study setting is Intermediate Hospital Oshakati.

**Methods:**

A quantitative cross-sectional design was adopted. Census sampling was employed, and the data were collected through the use of a structured, self-administered questionnaire. Data were analysed with the aid of the Statistical Package for the Social Sciences (SPSS) version 26.0 using frequencies and percentages, and Chi-square test of association.

**Results:**

Majority of the respondents were knowledgeable about prostate cancer except in questionnaire items 3, 12 and 13 in which they (71.8%, 82.4% and 94.7%, respectively) were not knowledgeable. In all the items of the questionnaire, majority of the respondents had positive attitudes towards prostate cancer screening. The percentages of the positive attitude range from 80% to 95% across all the items. There is no significant association between the respondents’ prostate cancer screening knowledge and all the respondents’ characteristics (age, level of education, marital status and religion), *p* > 0.05, respectively. There is no significant association between respondents’ prostate cancer screening attitude and all the respondents’ characteristics, *p* > 0.05, respectively.

**Conclusion:**

The survey respondents were knowledgeable about prostate cancer screening, except when it comes to the different methods of diagnosing prostate cancer, what happens to the prostate gland in prostate cancer and who should be screened for prostate cancer.

**Contribution:**

The results from this study can be used by the Ministry of Health and Social Services and its stakeholders to create a baseline data which help to develop appropriate preventative measures and awareness programmes. Furthermore, this study can be used to identify possible reasons for the late reporting of men for PCa screening and aid to inform the public on the need for early-seeking behaviour through screening.

## Introduction

Prostate cancer (PCa) is a considerable burden on health care globally.^1^ It is now one of the most common cancers found in many countries in the sub-Saharan Africa,^2^ with new cases expected to hit 1.7 million by 2030. It is expected that there will be 499 000 new deaths by the same year since 2012,^3^ particularly in the sub-Saharan Africa.

The incidence rate in the region was 34.3 per 100 000, while the mortality rate was 22.1 per 100 000.^4^ In Namibia, there were an estimated 1338 new PCa cases and 789 deaths in 2015. These numbers are expected to grow to 25 834 with 20 396 deaths by 2025.^5^ A 2020 report on the number of new cases of cancer indicates that PCa in Namibia forms 10.3% of all cancer types across both sexes and all ages. It also forms 23.8% of all cancers that occur in all ages for men.^6^

The rate of PCa and its related mortality in developing countries is increasing as a result of late diagnoses.^7^ It is expected to increase because of an increase in the ageing population and population growth. This means that preventive measures and early detection are of great importance.^8^ The diagnosis of PCa is performed by screening asymptomatic men through serum prostate-specific antigen (PSA) measurement, digital rectal examinations (DREs) and transurethral ultrasonography.^3,4,9^ But a positive diagnosis can only be confirmed through biopsies and microscopic examinations.^4^ These diagnoses are mostly carried out incidentally when doctors are looking for something else.^10^ However, PCa has not received enough public health attention in Namibia, thus the Namibian Cancer Society recommended that men older than 40 years should receive annual PCa screenings. This recommendation and other efforts did not improve the low screening rates in the country, however.^11^

Improved knowledge of PCa and its early detection are critical for reducing the number of late diagnoses and the high mortality rate caused by the disease across the African continent.^12^ Evaluating people’s knowledge and attitudes is also important for the creation of intervention plans that could be used to mitigate the PCa burden.^9^ Screening for PCa is crucial for the early detection of the condition and reducing the death rate.^3^ Thus, more needs to be done to boost the ongoing efforts to raise awareness about PCa in many African countries.^2^ One of the most important efforts involves obtaining baseline information from the vulnerable population, including their knowledge about, and attitudes towards, screening. This baseline information could serve as the basis for planning an appropriate intervention to raise people’s knowledge about PCa. The aim of this study was to assess the knowledge and attitudes about PCa screening among men over 50 years at Intermediate Hospital Oshakati.

## Research methods and design

### Study design and setting

A quantitative cross-sectional design was adopted. The study was conducted at the outpatient department (OPD) of Intermediate Hospital Oshakati, Oshana Region, which is in the northern part of Namibia. It is the largest and main referral hospital in the country, serving a catchment area of approximately 2m people encompassing the northern third of Namibia and a fair amount of the population of southern Angola. It has about 750 beds, treats close to 1m patients per year, delivers over 8000 babies per year and performs 8000+ surgical procedures per year with 5 operating theatres.

### Study population

The population of the study included all men over 50 years at the OPD of the hospital within the 3 months period of the study (August 2021 to October 2021).

### Sampling technique

The census sampling was employed, whereby all the accessible subjects who consented to the study formed the study population. Census sampling is when the entire population of the study is employed as a sample of the study.^13^ The census sampling was selected in this study because the population of the study was small. It is more advantageous to take the entire population to reduce the effect of attrition and non-response that could drastically reduce the sample if other sampling methods are used.

### Inclusion and exclusion criteria

The inclusion criterion was all men over 50 years with PCa attending Intermediate Hospital Oshakati, Namibia. However, participants who were not available during the period of data collection were excluded from the study.

### Instrument for data collection

The data for the study were collected with the use of a structured, self-administered, 22-item questionnaire that consisted of three sections: demographic characteristics, knowledge of PCa screening and attitude towards the screening. The nature of the questionnaire included yes or no questions. Content and face validity were used to ascertain the validity of the instrument. This is done through three experienced scholars who vetted the questionnaire. Advices, corrections and modifications from the scholars were effected accordingly. The questionnaire was piloted with 20 respondents who met the inclusion criteria but were not part of the sample. The pilot study provided insight into the likely problems that could be encountered during data collection. This included the most appropriate time to collect the data and the questions that were unclear to the respondents. Appropriate time that maximise the response was used and wordings and sentences commensurate to the respondents’ understanding were applied. The reliability of the questionnaire was found to be 0.76 calculated using Cronbachs’ alpha using the piloted data.

### Method of data analysis

With the aid of the Statistical Package for the Social Sciences (SPSS) version 26.0., the data were analysed using descriptive and inferential statistics. Data were coded as 1 = correct and 0 = wrong for knowledge, while for attitude 1 = positive attitude and 0 = negative attitude. The measuring scale for the level of knowledge was the mean score of ≤ 0.6, low knowledge; 0.61–0.80, moderate knowledge; and 0.81–1.00, high knowledge. The measuring scale for the level of attitude was the mean score of ≤ 0.6, negative attitude; 0.61–0.80, moderate attitude; and 0.81–1.00, positive attitude. This measuring scale was used as the respondents of the study were elderly people, with supposed related experiences. Thus, their supposed level of knowledge could have been better than younger people. Categorical variables were illustrated using frequencies and percentages. The Chi-square test of association was also used to determine the association between knowledge and respondents’ characteristics and between attitude and respondents’ characteristics. For all analyses, the statistical significance was set at the 0.05 level.

### Ethical considerations

Before conducting the study, institutional approval was sought from the University of Namibia Ethics and Research Committee (SoNREC 95/2020). Ethical approval was also obtained from the Executive Director of MoHSS, Research Unit (Ref: LKK 2020). The participants’ data were accessible to only the three researchers involved in the study. To ensure participants’ privacy, anonymity and confidentiality, participants’ names and all other identities were not included in the questionnaire. Access to data was strictly maintained to contribute to the quality of the study. Participants were also given the right to quit from the study at any point without having to explain themselves or receive penalties for doing so. Written informed consent was obtained from the participants individually.

## Results

Table 1 reveals that the respondents within the age bracket of 61–65 years had the largest percentage (37.4%), and the respondents within the age bracket of 56–60 years had the smallest percentage (12.2%). A primary education was the most prevalent level of education (37.4%), followed by a non-formal education (34.4%). The majority (73.3%) of the respondents were married. The majority (59.5%) of the respondents practised the Elcin religion.

**TABLE 1:** Respondents’ characteristics (*N* = 131).

Variables	Frequency (*n*)	Percentage (%)
**Age (years)**		
51–55	26	19.8
56–60	16	12.2
61–65	49	37.4
> 65	40	30.5
**Educational level**		
Non-formal	45	34.4
Primary	63	48.1
Secondary	18	13.7
Tertiary	5	3.8
**Marital status**		
Single	31	23.7
Married	96	73.3
Divorce	4	3.1
**Religion**		
Elcin	78	59.5
Anglican	20	15.3
Catholic	25	19.1
Others	8	6.1

Table 2 indicates that the majority of the respondents were knowledgeable about PCa, except regarding diagnosis, prostate cell changes and people to be screened (items 3, 12 and 13) for which they (71.8%, 82.4% and 94.7%, respectively) were not.

**TABLE 2:** Respondents’ knowledge of prostate cancer screening (*N* = 131).

Items	Correct		Wrong	
	*F*	%	*F*	%
1. Men aged 50 years and above should go for prostate cancer screening yearly	117	89.3	14	10.7
2. A digital rectal examination (DRE) is a type of prostate cancer screening	96	73.3	35	26.7
3. The only way to diagnose prostate cancer is through a prostate examination	37	28.2	94	71.8
4. For men without a prostate cancer diagnosis or symptoms that might indicate prostate cancer, does a PSA level measured at a particular age in men assist with determining the recommended interval to the next PSA test	113	86.3	18	13.7
5. Prostate-specific antigen (PSA) is a type of prostate cancer screening	111	84.7	20	15.3
6. A decrease in the flow or velocity of the urine stream is one of the signs of possible prostate cancer in men	102	77.9	29	22.1
7. Painful urination and ejaculation are some of the symptoms of prostate cancer	102	77.9	29	22.1
8. Blood in the urine or semen is one of the symptoms of prostate cancer	108	82.4	23	17.6
9. Difficulty in starting or stopping urination is one of the symptoms of prostate cancer	107	81.7	24	18.3
10. Sudden erectile dysfunction is among the symptoms of prostate cancer	67	51.1	64	48.9
11. Men aged 50 years and above are at risk of getting prostate cancer	121	92.4	10	7.6
12. Prostate cancer is caused when cells in the prostate become abnormal	23	17.6	108	82.4
13. Only those men with urinary symptoms should be screened	7	5.3	124	94.7

*F*, number; PSA, prostate-specific antigen.

Table 3 shows that in all the items of the questionnaire, the majority of the respondents had positive attitudes about PCa screening. The percentages of these positive attitudes ranged from 81.7% to 95.4% across all the items.

**TABLE 3:** Respondents’ attitude towards prostate cancer (*N* = 131).

Items	Positive		Negative	
	*F*	%	*F*	%
1. Men aged 50 and above should go for prostate cancer screening every 12 months	123	93.9	8	6.1
2. Men aged 50 and above should visit a health facility for prostate cancer screening when they experience backache	110	84.0	21	16.0
3. I visit a health facility for prostate cancer screening whenever I experience pain while urinating	119	90.8	12	9.2
4. I visit a health facility for prostate cancer screening whenever I observed blood in my urine	119	90.8	12	9.2
5. I visit a health facility for prostate cancer screening whenever I experience pain while ejaculating	107	81.7	24	18.3
6. Upon obtaining a positive result from a prostate cancer screening, I will undergo further screening every week if I don’t need to undergo treatment	125	95.4	6	4.6
7. Upon obtaining a negative result from a prostate cancer screening despite having symptoms, I will undergo further screening once a year at most	124	94.7	7	5.3
8. Upon obtaining a positive result from a prostate cancer screening, I will undergo further screening once a year if I am on prescribed Treatments	121	92.4	10	7.6
9. Upon obtaining a negative result from a prostate cancer screening despite having symptoms, I will undergo further screening twice a year at most	120	91.6	11	8.4

*F*, number.

Table 4 revealed that the majority (68.7%) of the respondents had moderate knowledge of PCa screening, while only 19.1% of the respondents had a high level. There is no significant relationship between respondents’ PCa screening knowledge and all the respondents’ characteristics (age, level of education, marital status and religion), *p* > 0.05, respectively.

**TABLE 4:** Association between knowledge of prostate cancer with respondents’ characteristics (*N* = 131).

Variable	Low knowledge		Moderate knowledge		High knowledge		*p*
	*F*	%	*F*	%	*F*	%	
**Age (years)**							0.45
51–55	3	18.80	16	17.7	7	28.0	
56–60	4	25.00	10	11.1	2	8.0	
61–65	4	25.00	38	42.2	7	28.0	
> 65	5	31.30	26	28.9	9	36.0	
Total	16	12.20	90	68.7	25	19.1	
**Educational level**							0.26
1	8	50.00	31	34.4	6	24.0	
2	8	50.00	41	45.6	14	56.0	
3	0	0.00	13	14.4	5	20.0	
4	0	0.00	5	5.6	0	0.0	
Total	16	12.20	90	68.7	25	19.1	
**Marital status**							0.12
Married	2	12.50	27	30.0	2	8.0	
Single	14	87.50	60	66.7	22	88.0	
Divorce	0	0.00	3	3.3	1	4.0	
Total	16	12.20	90	68.7	25	19.1	
**Religion**							0.50
Elcin	10	62.50	49	54.4	19	76.0	
Anglican	3	18.75	15	16.7	2	8.0	
Catholic	2	12.50	19	21.1	4	16.0	
Others	1	6.25	7	7.8	0	0.0	
Total	16	12.20	90	68.7	25	19.1	

Note: 1 = Non-formal, 2 = Primary, 3 = Secondary, 4 = Tertiary.

*F*, number.

Table 5 indicates that 48.9% of the respondents had a moderate attitude towards the PCa screening, while 49.6% of the respondents had a positive attitude. There is no significant association between respondents’ PCa screening attitude and all the respondents’ characteristics, *p* > 0.05, respectively.

**TABLE 5:** Association between respondents’ attitude towards prostate cancer with their characteristics (*N* = 131).

Variable	Negative attitude		Moderate attitude		Positive attitude		*p*
	*F*	%	*F*	%	*F*	%	
**Age (years)**							0.41
51–55	0	0.0	14	21.90	12	18.5	
56–60	1	50.0	7	10.90	8	12.3	
61–65	1	50.0	27	42.20	21	31.3	
> 65	0	0.0	16	25.00	24	36.9	
Total	2	100.0	64	100.00	65	100.0	
**Educational level**							0.62
1	1	50.0	22	34.40	22	33.8	
2	1	50.0	3	50.00	30	46.2	
3	0	0.0	6	9.40	12	18.5	
4	0	0.0	4	6.25	1	1.5	
Total	2	100.0	64	100.00	65	100.0	
**Marital status**							0.22
Single	0	0.0	18	28.10	13	20.0	
Married	2	100.0	46	71.90	48	69.2	
Divorce	0	0.0	0	0.00	4	6.2	
Total	2	100.0	64	100.00	65	100.0	
**Religion**							0.86
Elcin	1	50.0	36	56.30	41	63.1	
Anglican	1	50.0	12	18.80	2	18.5	
Others	0	0.0	5	7.80	3	4.6	
Total	2	100.0	4	100.00	65	100.0	

Note: 1 = Non-formal, 2 = Primary, 3 = Secondary, 4 = Tertiary.

*F*, number.

## Discussion

The results of this study show that the highest percentage of respondents were over 60 years. Thus, older men consent to studies about PCa more than others. This finding agrees with a study in Nigeria in which the majority of the respondents were found to be in their old age (60–69 years and 80+ years), at 42.8% and 11.3%, respectively.^14^ As the majority of the respondents were old, it was not surprising that the majority only had a primary education or non-formal education, as Western education was not as common when they were young. This finding is as per a study by Gift et al.,^9^ in which 80.5% of the respondents had no formal education or had only a primary school education. This is not surprising as the two studies were hospital-based and have a common subcontinent area of the studies, the South African subcontinent. It was expected that the majority of the respondents would be Christians (Elcin, Anglican or Catholic), as the area in which the hospital is located is predominantly Christian. As with any study that only involves adult subjects, especially those in middle and old age, the respondents of this study were mostly married.^1,9,15^

The respondents demonstrated a high level of knowledge regarding most of the items on the questionnaire; however, few got it right when they were asked if prostate examinations are the only way to diagnose PCa. This is contrary to a study in Saudi Arabia, in which 41.8% of the respondents were found to be knowledgeable about PSA levels,^16^ as well as in Italy, in which 72.7% of the respondents were found to be knowledgeable^8^ about PSA levels. The differences between the findings of the two studies with the findings of this study could be because of differences in the areas of the studies. The study in Saudi Arabia was conducted in primary care centres and among men aged between 40 years and 90 years. The study in Italy was conducted among selected public school students’ fathers. However, this study was conducted in a tertiary hospital and among men aged 50 years and above. These differences between the studies could be the basis for the differences in their findings. In accordance with the findings of this study, a study in Nigeria found that the majority of the respondents (58%) were not aware of PCa screening.^7^ The two studies were conducted in the African continent, which could be the reason for the similarities in this finding.

Most of the respondents to this study did not correctly answer the question regarding if PCa occurs when cells in the prostate become abnormal. In accordance with this finding, a study in Malaysia identified only 46.21% to have answered correctly that any prostatic enlargement is cancer.^17^ The similarity in this finding between the two studies exists despite the fact that the two studies were conducted in different continents and the two study areas grossly differ. The study in Malaysia was conducted in a mall among all adults. Moreover, the majority of the respondents in this study did not correctly answer whether only those men with urinary symptoms should be screened. In parallel to this finding, a study in India revealed that only 3.59% of the respondents answered correctly as to whether only those with problems urinating should be screened.^18^ Thus, the respondents from the two studies were highly unknowledgeable regarding methods of prostate examination, the state of prostate in PCa and the people who need to be screened for PCa. The implication of these findings is therefore that policymakers and healthcare professionals need to put more effort into educating people about all aspects of PCa. The two studies shared the common ground of being hospital-based studies and considered men over 50 years as the population of the study.

When looking at people’s general knowledge of PCa, contrary to this study, Yee et al.^17^ found that the majority of their respondents had adequate knowledge about PCa screening. Ahmed et al.,^18^ however, found similarly low levels of awareness about PCa screening (47.48%) among the respondents of their study. Furthermore, a study by Gift et al.^9^ revealed that out of 200 participants, just 29% expressed knowledge about PCa, with the majority (63.8%) having little knowledge. The implication is that those with little knowledge of PCa and/or PCa screening are not getting screened. This calls for concerned stakeholders to intensify their efforts to educate citizens about PCa and screening.

The findings of this study revealed no significant association between the respondents’ characteristics (age, level of education, marital status and religion) and their knowledge of PCa screening (*p* > 0.05). This finding is in accordance with a study that assessed the knowledge and practices of PCa screening among men in the eastern region of Ghana, where it was found that age group, marital status, educational attainment and religious affiliation were not significantly associated with people’s knowledge about PCa (*p* > 0.05).^19^ The two studies differ in the sense that this study was hospital based, while the study in Ghana was community based. The study in Ghana involved all men, while this study involved only those above 50 years of age. This means that awareness of PCa and PCa screening should be given to all men, irrespective of their age, educational level, marital status or religion. However, contrary to this study, Gift et al.^9^ found that participants with secondary or tertiary education had more knowledge about PCa than those with less education (*p* < 0.001). Also, in the same study, participants older than 60 years were more knowledgeable than those under 60 years (*p* < 0.05). This difference may be because of differences in the two study areas and differences in the categorisation of the respondents’ ages.

The majority of the respondents had either positive or moderate attitudes towards PCa screening, with an insignificant percentage having a negative attitude. All the individual items constitute a positive attitude (81% – 95%) towards PCa screening. Parallel to this study, another study by Musalli et al.^16^ found that the majority of the respondents had a good attitude regarding PCa screening (71%). In contrast, however, research on people’s knowledge and attitude towards PCa and screening practices among men in Saudi Arabia found that over half of the respondents (53.1%) had a negative attitude towards PCa and screening.^10^ This variation could be because of differences in the area of the study, the instrument for data collection and/or the measuring scale. Also, this study’s population comprised of participants above 50 years, while the study in Saudi Arabia comprised of participants above 40 years. The findings of this study show that men have good intentions towards PCa screening, and thus, little effort should be needed to make them utilise the services pertaining to PCa screening.

No significant association was found between the respondents’ characteristics (age, level of education, marital status and religion) and their attitudes towards PCa screening. In the same vein, Alothman et al.^10^ found no significant association between their respondents’ ages, levels of education and marital status with PCa and PCa screening (*p* > 0.05). Contrary to the findings of these two studies, however, Musalli et al.^16^ found a significant association between age and patients’ attitudes regarding prostate examinations (*p* = 0.000), yet they also found no association between education and patients’ attitudes regarding prostate examination (*p* = 0.506). A lack of association between the respondents’ characteristics and their attitudes towards PCa screening indicates that men should not be discriminated against on the basis of age, education, marital status or religion when raising awareness of PCa.

## Conclusion

This study investigated the knowledge and attitudes about PCa screening among men over 50 years at Intermediate Hospital Oshakati. The respondents were knowledgeable about PCa screening, except when it came to the different methods of diagnosing PCa, what happens to the prostate gland when there is PCa and who should be screened for PCa. Cumulatively, about one-tenth of the respondents were found to have low levels of knowledge regarding PCa screening. There was largely a positive attitude regarding PCa across all the items of the questionnaire, and there were no significant associations between the respondents’ characteristics and their knowledge of PCa screening and attitude towards PCa screening. There is need for more efforts in educational and awareness campaigns about PCa and/or PCa screening. There should be policies that provide PCa screening public campaign and education to the public, and PCa screening accessible and affordable.

### Limitations of the study

The use of only one hospital and limited number of participants are the limitations of this study. The study’s findings may be specific to the Oshana region in Namibia, as data were collected only from respondents in this area. Therefore, the findings may not be representative of the entire country or other regions with different contexts and healthcare systems. Two or more hospitals could have provided more samples for the study. This is a factor that may affect the generalisability of the findings. Triangulation of data from multiple sources or methods could enhance the validity of the findings. The study could benefit from incorporating perspectives from nurses, doctors and other stakeholders involved in caring for patient with PCa.

### Implications for practice and policy

The findings of this study revealed that those men with little knowledge of PCa and/or PCa screening are most likely not being screened. It indicates the need for putting on more efforts in educating and passing awareness about PCa and/or PCa screening. It is also clear from the findings of this study that all men need to be aware and educated about PCa and PCa screening irrespective of their age, educational level, marital status or religion. High positive attitude about PCa and/or PCa screening found in this study shows that educating the citizens and passing awareness on PCa and/or PCa screening would bring about great changes in utilising the services pertaining to PCa screening. Therefore, policymakers at all levels of government should provide a policy that will make public campaign and education about utilisation of the services pertaining to PCa screening mandatory. The policy should also make the provision of those services at all hospital levels compulsory and affordable.

### Future research directions

More studies should be performed on factors influencing the knowledge and attitude of men towards PCa and PCa screening, as well as factors influencing the acceptance of PCa screening.
